# Pharmacogenomics in diabetes: outcomes of thiamine therapy in TRMA syndrome

**DOI:** 10.1007/s00125-018-4554-x

**Published:** 2018-02-15

**Authors:** Abdelhadi M. Habeb, Sarah E. Flanagan, Mohamed A. Zulali, Mohamed A. Abdullah, Renata Pomahačová, Veselin Boyadzhiev, Lesby E. Colindres, Guillermo V. Godoy, Thiruvengadam Vasanthi, Ramlah Al Saif, Aria Setoodeh, Amirreza Haghighi, Alireza Haghighi, Yomna Shaalan, International Neonatal Diabetes Consortium, Andrew T. Hattersley, Sian Ellard, Elisa De Franco

**Affiliations:** 1 Paediatric Department, Prince Mohammed bin Abdulaziz Hospital, National Guard Ministry, P.O. Box 40740, Al Madinah, 41511 Kingdom of Saudi Arabia; 2 Institute of Biomedical and Clinical Science, University of Exeter Medical School, Royal Devon and Exeter Hospital, Barrack Road, Exeter, EX2 5DW UK; 3 Paediatric Department, College of Medicine, Taibah University, Madinah, Kingdom of Saudi Arabia; 4 Paediatric Department, Khartoum University, Khartoum, Sudan; 5 Department of Paediatrics, Charles University, Medical Faculty and University Hospital Pilsen, Pilsen, Czech Republic; 6 Medical University, Varna, Bulgaria; 7 Hospital María De Especialidades Pediatricas, Tegucigalpa, Honduras; 8 Kanchi Kamakoh Child Trust Hospital, Chennai, India; 9 Paediatric Department, Maternity and Children’s Hospital, Dammam, Kingdom of Saudi Arabia; 10 Growth & Development Research Centre, University of Tehran, Medical Sciences, Tehran, Iran; 11 Toronto General Hospital, University of Toronto, Toronto, ON Canada; 12 Department of Genetics and Medicine, Harvard Medical School, Boston, MA USA; 13 Broad Institutes of Harvard and MIT, Cambridge, MA USA; 14 Partners HealthCare Laboratory for Molecular Medicine, Cambridge, MA USA; 15 Faculty of Medicine, Cairo University, Cairo, Egypt

**Keywords:** Pharmacogenomics, Thiamine therapy, TRMA-related diabetes, Vitamin B_1_

## Abstract

**Aims**/**hypothesis:**

Diabetes is one of the cardinal features of thiamine-responsive megaloblastic anaemia (TRMA) syndrome. Current knowledge of this rare monogenic diabetes subtype is limited. We investigated the genotype, phenotype and response to thiamine (vitamin B_1_) in a cohort of individuals with TRMA-related diabetes.

**Methods:**

We studied 32 individuals with biallelic *SLC19A2* mutations identified by Sanger or next generation sequencing. Clinical details were collected through a follow-up questionnaire.

**Results:**

We identified 24 different mutations, of which nine are novel. The onset of the first TRMA symptom ranged from birth to 4 years (median 6 months [interquartile range, IQR 3–24]) and median age at diabetes onset was 10 months (IQR 5–27). At presentation, three individuals had isolated diabetes and 12 had asymptomatic hyperglycaemia. Follow-up data was available for 15 individuals treated with thiamine for a median 4.7 years (IQR 3–10). Four patients were able to stop insulin and seven achieved better glycaemic control on lower insulin doses. These 11 patients were significantly younger at diabetes diagnosis (*p* = 0.042), at genetic testing (*p* = 0.01) and when starting thiamine (*p* = 0.007) compared with the rest of the cohort. All patients treated with thiamine became transfusion-independent and adolescents achieved normal puberty. There were no additional benefits of thiamine doses >150 mg/day and no reported side effects up to 300 mg/day.

**Conclusions/interpretation:**

In TRMA syndrome, diabetes can be asymptomatic and present before the appearance of other features. Prompt recognition is essential as early treatment with thiamine can result in improved glycaemic control, with some individuals becoming insulin-independent.

**Data availability:**

*SLC19A2* mutation details have been deposited in the Decipher database (https://decipher.sanger.ac.uk/).

**Electronic supplementary material:**

The online version of this article (10.1007/s00125-018-4554-x) contains peer-reviewed but unedited supplementary material, which is available to authorised users.


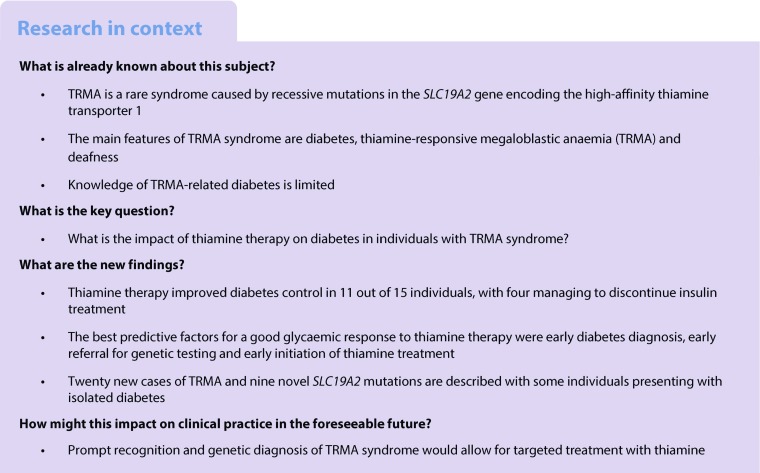


## Introduction

Thiamine-responsive megaloblastic anaemia (TRMA) syndrome, also known as Roger’s syndrome, is a rare congenital disease characterised by a triad of cardinal features: thiamine-responsive anaemia, diabetes and deafness [1–4]. It is caused by recessively inherited mutations in the *SLC19A2* gene encoding the high-affinity thiamine transporter 1 (THTR1) [5].

Thiamine (vitamin B_1_) is an essential vitamin for normal beta cell function and a co-factor for a number of intracellular enzymes in various tissues. At physiological levels, thiamine is mainly transported into the cells through two high-affinity transporters, THTR1 and THTR2, that are encoded by the *SLC19A2* and *SLC19A3* genes, respectively. At high concentrations thiamine can cross the cell membrane through passive diffusion [6]. Biallelic *SLC19A2* mutations result in non-functional THTR1; however, most tissues can compensate through THTR2. Studies in humans and animal models have shown different tissue expression patterns for the two thiamine transporters, with THTR2 being absent or minimally expressed in pancreatic endocrine cells, bone marrow and cochlear cells [7–9]. This indicates that THTR1 is the main thiamine transporter in these three tissues and explains the presence of deafness, diabetes and anaemia in individuals with TRMA syndrome [10–13].

Onset of TRMA syndrome is usually between infancy and adolescence, with any of the three classical features. In some individuals additional features such as thrombocytopenia, neurological symptoms, cardiac anomalies, visual disturbances and short stature have also been reported [14–17].

To date, more than 40 different *SLC19A2* mutations have been identified in less than 60 families [18–23]. The majority of these have been described in individual case reports or small series and the focus has been mainly on the haematological manifestations of the condition. Data on the clinical characteristics and long-term follow-up of diabetes in these individuals is, however, limited.

With improved access to genetic testing, we expect that more individuals with *SLC19A2* mutations will be referred to diabetes clinics. Considering the limited knowledge on this condition and the paucity of long-term follow-up data, we aimed to investigate the genotypic and phenotypic features and long-term effects of thiamine treatment on glycaemic control in an international series of individuals with diabetes caused by *SLC19A2* mutations.

## Methods

The study was conducted in accordance with the Declaration of Helsinki with informed consent to perform genetic analysis given by individuals with TRMA syndrome or their parents/guardians.

Individuals with TRMA syndrome were identified from the Exeter monogenic diabetes database with initial clinical details provided by the referring clinicians via a standard request form (available at www.diabetesgenes.org). Further information on clinical follow-up and response to thiamine therapy were obtained from the clinicians through a follow-up questionnaire.

Thiamine responders were defined as individuals for whom the total daily insulin dose was decreased by ≥10% without a deterioration in HbA_1c_ or HbA_1c_ was reduced by ≥11 mmol/mol (1%) while on a similar insulin dose within 6 months of starting thiamine therapy. Clinicians were specifically asked what they thought was the maximum dose of thiamine needed to achieve response.

### Genetic testing

Genetic testing was performed in Exeter (UK) for all of the individuals in our cohort, except 20.1, who was tested at Dammam Children’s Hospital, Kingdom of Saudi Arabia. DNA was extracted from peripheral blood using standard methods. Direct sequencing of the *KCNJ11*, *ABCC8*, *INS* and *EIF2AK3* genes was initially conducted in all individuals diagnosed with diabetes before 6 months of age according to previously described methods [24]. If the phenotype was suggestive of TRMA syndrome, direct sequencing of *SLC19A2* was performed. The six coding exons of the *SLC19A2* gene were screened by Sanger sequencing as described previously [16] (primers available on request). Sequencing reactions were run on an ABI3730 capillary machine (Applied Biosystems, Warrington, UK) and analysed using Mutation Surveyor v3.98 (SoftGenetics, State College, PA, USA) (*SLC19A2* nucleotide reference NM_006996.2). The bioinformatics tools SIFT, PolyPhen-2 and Align GVGD were accessed through the ALAMUT Visual software version 2.7.1 (Interactive Biosoftware, Rouen, France) to predict the effect of novel variants on the SLC19A2 protein. Mutation testing of family members was performed when DNA was available.

In individuals 19.1 and 21.1, who had isolated neonatal diabetes at the time of referral, the *SLC19A2* mutation was detected via targeted next generation sequencing of all known neonatal diabetes genes, as previously described [25]. These were the only two individuals with homozygous *SLC19A2* mutations identified in a cohort of 921 individuals with diabetes diagnosed before the age of 6 months tested with this technique in the Exeter molecular genetics laboratory.

### Statistical analysis

The Mann–Whitney *U* test was used to compare individuals who showed improved glycaemic control on thiamine therapy vs those who showed no improvement. Data are expressed as median (IQR). A *p* < 0.05 was considered to be statistically significant.

## Results

We investigated 32 individuals (27 families) from 17 countries. Consanguinity was reported in 13 families. At the time of referral for genetic testing, the age of the individuals in the cohort ranged from 3 months to 28 years (median age 4 years [IQR 1–9]). We describe 20 previously unreported individuals with TRMA syndrome and present follow-up data for 12 individuals reported previously [16, 26].

### Genotype

**Fig. 1:**
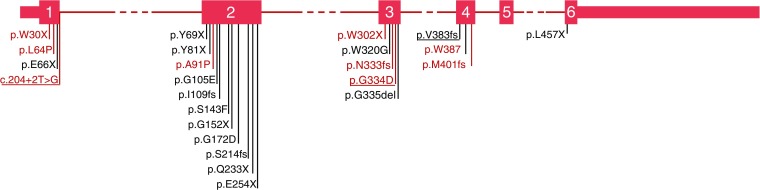
Schematic representation of the *SLC19A2* gene with mutations identified in our cohort. Novel mutations are highlighted in red. Compound heterozygous mutations are underlined

Twenty-four different *SLC19A2* mutations were identified in the 32 individuals from 27 families, confirming a genetic diagnosis of TRMA syndrome. Four individuals from three families were compound heterozygotes for *SLC19A2* mutations and the remaining participants had a homozygous mutation (Table 1, Fig. 1 and electronic supplementary material [ESM] Table 1).

**Table 1:** Mutation description and phenotype of study participants

Individual	Mutation description	Clinical features and age at onset			
		Diabetes	Anaemia	Deafness	Other reported features
1.1	p.L64P/p.L64P^a^	35 weeks	4 years	4 years	–
1.2	p.L64P/p.L64P^a^	8 years	7.5 years	3 years	–
1.3	p.L64P/p.L64P^a^	6 years	6 years	4 years	–
2.1	p.W30*/p.W30*^a^	+; age NA	+; age NA	+; age NA	–
3.1 [16]	p.E66*/p.E66*	32 weeks	1 day	–	Squint, spastic quadriplegia, talipes, cerebral atrophy
4.1	p.E66*/p.E66*	+; age NA	+; age NA	+; age NA	–
5.1 [16]	p.Y79*/p.Y79*	6 months	6 months	6 months	Meningoencephalitis at 6 months, hypotonia, developmental delay, mitral tricuspid abnormality
6.1 [20]	p.Y81*/p.L457*	20 months	7 years	20 months	Retinitis pigmentosa at 36 months
6.2 [20]	p.Y81*/p.L457*	27 months	–	8 months	Macrocytosis at 27 months, pigmentary retinal alteration, reduced optic nerve thickness
7.1	p.A91P/p.A91P^a^	43 weeks	43 weeks	43 weeks	–
8.1	p.G105E/p.G105E	2 years	2 years	2 years	Thrombocytopenia, macular degeneration, nystagmus
9.1 [16]	p.I109fs/p.I109fs	4 months	3 months	6 months	Supraventricular tachycardia, cardiomyopathy, stroke at 2 years, speech delay
10.1	p.I109fs/p.I109fs	2.3 years	3 years	2 years	Speech delay; died at 7 years old of DKA
10.2	p.I109fs/p.I109fs	3 months	3 months	8 months	Nystagmus
11.1 [16]	p.S143F/p.S143F	6 weeks	4 weeks	14 months	Nystagmus, myopia, short stature, developmental delay
12.1	p.S143F/p.S143F	3 years	2 years	4 years	–
13.1	p.G152*/p.G152*	+; age NA	+; age NA	+; age NA	–
14.1	p.G172D/p.G172D	7 months	6 months	–	Died at 6 months
15.1	p.S214fs/p.S214fs	12 weeks	12 weeks	12 weeks	–
16.1 [26]	p.Q233*/p.Q233*	8 months	8 months	10 months	Thrombocytopenia, short stature and developmental delay
17.1 [26]	p.Q233*/p.Q233*	16 months	3 months	1 day	Seizures at 2 years, stroke at 16 months
17.2 [26]	p.Q233*/p.Q233*	2 years	1 year	1 day	Developmental delay, short stature
18.1 [26]	p.Q233*/p.Q233*	7 years	5 years	1 day	Developmental delay
19.1	p.E254*/p.E254*	5 months	–	6 months	Speech delay
20.1	p.W302*/p.W302*^a^	18 months	18 months	2 years	Myopia
21.1	p.W320G/p.W320G	12 weeks	–	–	–
22.1	p.N333fs/p.N333fs^a^	3.5 years	3.5 years	4.5 years	Developmental delay
23.1	c.204+2T>G^a^/p.G334D^a^	2 years	2 years	3 years	Atrial ectopic tachycardia
24.1 [16]	p.G334D/p.V383fs	7 months	5 months	5 years	Speech delay, maculopathy, hyperpigmentation of left upper limb and skin
25.1 [22]	p.G335del/p.G335del	3 years	2 years	2 years	Patent ductus arteriosus, myopia
26.1	p.M401fs/p.M401fs^a^	20 weeks	20 weeks	+; age NA	Thrombocytopenia
27.1	p.W387*/p.W387*^a^	20 weeks	8 weeks	1 year	Thrombocytopenia, mild leucopenia, ASD

^a^Novel mutation

ASD, atrial septal defect; del, deletion; fs, frameshift; NA, not available. The * symbol indicates a Stop codon

Nine of the mutations identified were novel (three missense, three nonsense, two frameshift and one splice site mutation). The three novel missense mutations (p.Leu64Pro, p.Ala91Pro and p.Gly334Asp) affect residues highly conserved across species and in silico investigations predict these mutations to be pathogenic. Only p.Gly334Asp was listed in the Exome Aggregation Consortium (ExAC) database (minor allele frequency (MAF) 1.499 × 10^−05^; http://exac.broadinstitute.org/, accessed November 2016) in a single heterozygous individual of European ancestry. The two frameshift (c.993_996dup and c.1201_1202del) and three nonsense (p.Trp30*, p.Trp302* and p.Trp387*) mutations are predicted to cause the insertion of a premature stop codon, which probably results in mRNA degradation via nonsense mediated decay. The splicing mutation (c.204+2T>G) is predicted to abolish the splicing donor site in intron 1 and cause aberrant splicing.

### Phenotype

**Fig. 2:**
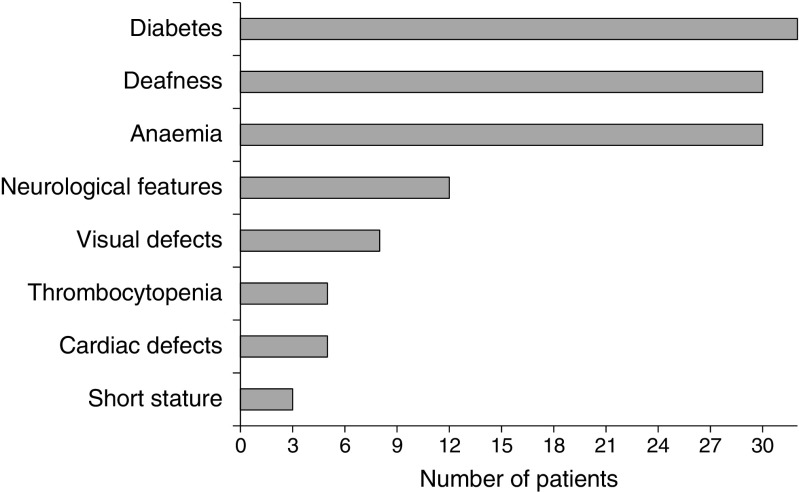
Frequency of the clinical features in the study participants

**Fig. 3:**
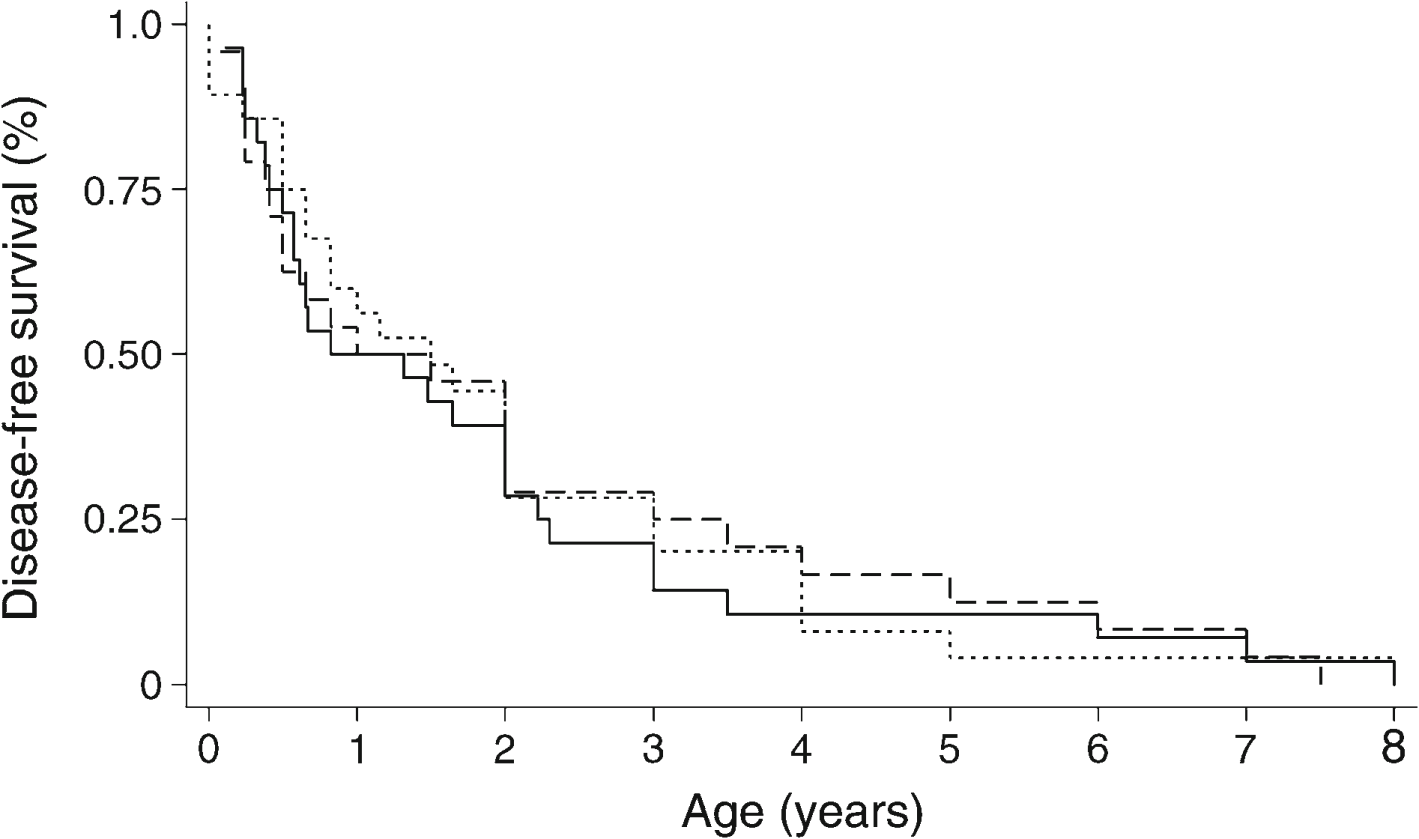
Kaplan–Meier plot showing the probability of developing each TRMA clinical feature. Solid line, diabetes; dotted line, deafness; dashed line, anaemia

The clinical characteristics and demographics of each individual are summarised in Table 1, Figs 2, 3 and ESM Table 1. The exact age at presentation was not available for three individuals (2.1, 4.1 and 13.1). In the remaining 29 participants, the age at diagnosis of the first symptom ranged from birth to 4 years (median 26 weeks [IQR 12–104]). Variable age at onset was observed between individuals with the same mutation, including siblings, indicating intra-familial variability.

Three individuals (5.1, 7.1 and 15.1) presented with the classical triad of TRMA syndrome, whilst ten were initially diagnosed with diabetes and anaemia with deafness subsequently confirmed on hearing test. The remaining individuals developed these clinical features in variable order.

All 32 individuals had diabetes, with age at onset ranging from 6 weeks to 8 years (median 43 weeks [IQR 21–116]). Diabetes was diagnosed in the first year of life in 15 individuals, with nine of them being diagnosed before the age of 6 months, fulfilling diagnostic criteria for neonatal diabetes [24]. In three individuals (1.1, 19.1 and 21.1) diabetes was diagnosed before appearance of the other clinical features, whilst in 11 individuals hyperglycaemia was the presenting symptom along with anaemia and/or deafness. Three children presented with diabetic ketoacidosis (DKA), 17 with symptomatic hyperglycaemia and in the remaining 12 individuals hyperglycaemia was an incidental finding. Islet cell autoantibodies were measured in three participants and the tests were negative. C-peptide was measured in four individuals, at least 1 year after starting insulin, and the level was <0.165 nmol/l for all of them. After a diagnosis of diabetes, all patients started insulin therapy and all except four are still on insulin with variable glycaemic control. Recurrent DKA episodes were reported for two individuals and one child died of DKA in a remote hospital during a family holiday.

Anaemia was documented in all except two individuals: individual 19.1 who still has normal haemoglobin (Hb) and mean corpuscular volume (MCV) at the age of 3.9 years; and individual 6.2 who was reported to have macrocytosis in the presence of normal Hb [21]. In the other participants in the cohort anaemia presented between birth and 7.5 years (median 47 weeks [IQR 14–155]) and initial Hb levels ranged from 43–75 mg/l (normal range 115–150 mg/l). In 18 individuals anaemia was the first presenting symptom, either in isolation (individuals 3.1, 9.1, 11.1, 12.1, 14.1, 24.1 and 27.1) or in combination with other features. For the rest of the cohort, anaemia developed after the onset of diabetes. All individuals with anaemia needed multiple blood transfusions prior to starting thiamine therapy.

All except two individuals (3.1 and 14.1) were reported to have sensorineural deafness. This was diagnosed between birth and 4.5 years of age (median 60 weeks [IQR 26–156]). Deafness was the presenting feature in seven individuals. In individuals 17.1, 17.2 and 18.1 it was detected soon after birth and in individuals 1.2, 1.3, 6.2 and 10.1 it was diagnosed up to 4.5 years before the appearance of the other features. In the remaining individuals deafness was detected by hearing tests following the appearance of other clinical features. Two individuals (8.1 and 27.1) had cochlear implantation, while the rest have hearing aids. Regarding the two individuals with no reported deafness, individual 14.1 died at the age of 15 months and individual 3.1 has severe neurodevelopmental delay with no follow-up audiogram since initial presentation.

Additional clinical features, other than anaemia, diabetes and deafness, were reported in 22 individuals (Table 1 and Fig. 2). These included neurological deficit/developmental delay (37.5%), visual defects (25.0%), cardiac anomalies (15.6%), short stature (9.4%) and thrombocytopenia (12.5%).

### Follow-up and response to thiamine therapy

Follow-up data was available for 16 individuals (Table 2). This includes individual 14.1 who died at the age of 15 months following a viral infection before starting thiamine therapy. For the other 15 participants, thiamine therapy was commenced following the genetic diagnosis. The age at starting thiamine treatment ranged from 6 months to 14 years (median 24 years [IQR 12.5–76]) and the maximum dose ranged between 25 and 300 mg/day. However, none of the clinicians observed any additional benefits of thiamine doses >150 mg/day. The duration of thiamine therapy was between 2 and 17.5 years (median 4.7 years) giving a total period of 103.5 treatment years.

**Table 2:** Long-term follow-up data and response to thiamine in 16 individuals

Individual	Presenting age and symptoms	Pre-treatment HbA_1c_, mmol/mol (%)	Thiamine therapy				Current condition^a^			
			Starting age	Maximum daily dose (mg)	Duration	Benefit on diabetes	Age	Insulin (U kg^−1^ d^−1^)	Mean HbA_1c_ mmol/mol (%)	Comments and prognosis
8.1	2 y; diabetes, anaemia	106.6 (11.9)	2 y	250	12 y	Yes^b^	14 y	1.0	76.0 (9.1)	Normal growth and puberty; cochlear implant
9.1	4 m; anaemia	95.6 (10.9)	6 m	150	7.5 y	Yes^b^	8 y	0.5	63.9 (8.0)	Normal growth; stroke while on thiamine
10.1	2 y; deafness	90.2 (10.4)	3 y	200	4 y	Yes^b^	Died at 7 y	0.6	–	Died of DKA
10.2	3 m; anaemia	82.5 (9.7)	1 y	100	3 y	Yes^b^	4 y	0.3	85.8 (7.0)	Normal growth; nystagmus
11.1	4 w; anaemia	95.6 (10.9)	14 m	200	4.7 y	Yes^b^	6 y	0.9	73.8 (8.9)	Normal growth; speech delay, nystagmus and myopia
14.1	6 m; anaemia	55.2 (7.2)	–	–	–	–	Died at 15 m	1.0	–	Died of viral diarrhoea before thiamine therapy
16.1	8 m; anaemia, diabetes	73.8 (8.9)	8 m	100	3 y	Off insulin	3.7 y	–	34.4 (5.3)	Off insulin after thiamine therapy, normal growth
17.1	16 m; deafness	70.5 (8.6)	13 y	300	15 y	No	28 y	1.3	70.5 (8.6)	Short stature; normal puberty; seizure, stroke and developmental delay
17.2	2 y; deafness	58.5 (7.5)	14 y	300	17.5 y	No	31.5 y	1.5	58.5 (7.5)	Normal stature and puberty; developmental delay
18.1	5 y; deafness	66.1 (8.2)	13 y	300	13 y	No	17.9 y	1.4	66.1 (8.2)	Normal stature and puberty; developmental delay
19.1	5 m; diabetes	63.9 (8.0)	13 m	150	2.8 y	Off insulin	3.9 y	–	33.3 (5.2)	Normal growth; never had anaemia
20.1	18 m; diabetes, anaemia	74.9 (9.0)	3 y	150	8 y	Off insulin for 5 y	11 y	0.5	65 (8.1)	Normal growth; restarted on insulin recently
22.1	3.5 y; diabetes, anaemia	74.9 (9.0)	4.5 y	100	3 y	No	7.5 y	0.5	74.9 (9.0)	Developmental delay
23.1	2 y; diabetes, anaemia	77 (9.2)	2 y	50	2 y	Yes^b^	4 y	0.6	49.7 (6.7)	Normal growth
24.1	7 m; anaemia	50.8 (6.8)	8 y	50	5 y	Yes^b^	13 y	0.9	49.7 (6.7)	Normal growth and puberty
27.1	3 m; pancytopenia	91.3 (10.5)	9 m	100	3 y	Off insulin	4 y	–	37.7 (5.6)	Normal growth and development; cochlear implant; catheter closure for atrial septal defect

^a^All participants with anaemia on thiamine therapy had normal Hb without blood transfusion; however, deafness did not respond to thiamine

^b^Total daily insulin dose decrease of ≥10% without a deterioration in HbA_1c_, or HbA_1c_ reduction of ≥11 mmol/mol (1%) while on a similar insulin dose

d, day; m, months; w, weeks; y, years

**Fig. 4:**
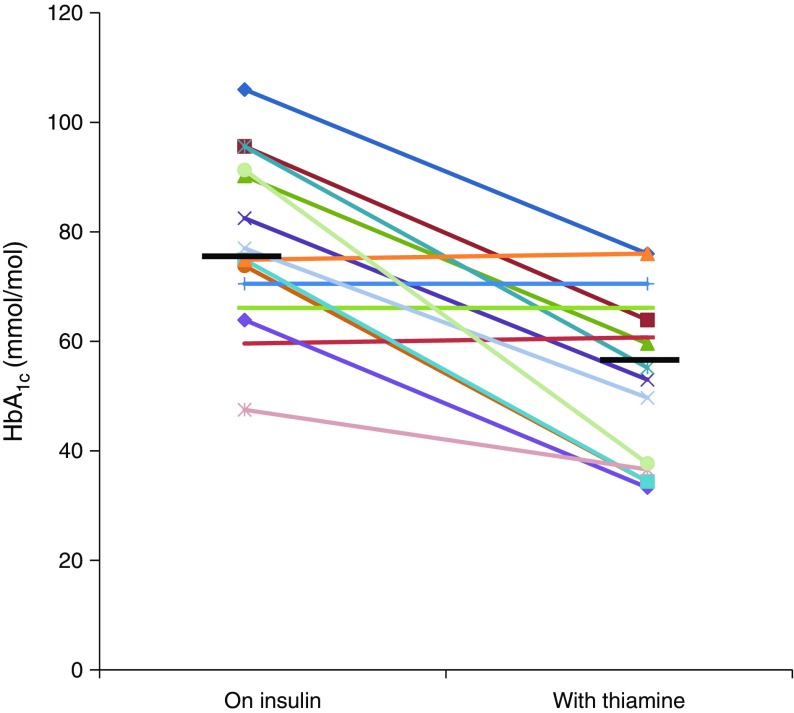
HbA_1c_ before and after starting thiamine therapy for the 15 individuals with long-term follow-up data. The horizontal black bars represent the median

A clear benefit of thiamine therapy on diabetes control was observed in 11 out of 15 patients. Seven individuals achieved improved HbA_1c_ levels, at least for the following 6 months (Fig. 4), with reduced insulin requirement, and four managed to become insulin-independent. Participants 16.1, 19.1 and 27.1 have been off insulin since starting thiamine at the ages of 8 months, 13 months and 9 months, respectively. Individual 20.1 (currently aged 11 years) has recently started to require insulin again following a period of 5 years of insulin independence on thiamine treatment alone. The 11 patients who showed a glycaemic benefit from thiamine therapy (responders) had a significantly younger age at diagnosis of diabetes (*p* = 0.042), were referred for genetic testing earlier (*p* = 0.01) and had started thiamine treatment earlier (*p* = 0.007) compared with the non-responder group (Table 3). Our study did not detect a significant difference in sex, age at onset of the first TRMA symptom, thiamine dose or duration of therapy between the responder and non-responder groups (Table 3).

**Table 3:** Analysis of demographic and clinical factors associated with response to thiamine

Variable	Responders (*n* = 11)	Non-responders (*n* = 4)	*p* value
Female sex	7	3	0.749
Median age at first symptom (months)	5	1	0.166
Median age at diabetes diagnosis (months)	7	33	0.042*
Median age at starting thiamine (months)	14	158	0.007**
Median age at referral for genetic testing (months)	12	77	0.010**
Maximum thiamine dose (mg/day)	250	300	0.062
Median duration of thiamine therapy (years)	4	14	0.065

*p < 0.05, **p ≤ 0.01

In all individuals with anaemia, Hb rose to normal levels within 6–8 weeks of starting thiamine therapy and all living participants are currently transfusion-independent. Similarly, participants with thrombocytopenia have maintained normal platelet counts while on thiamine. No impact of thiamine therapy was reported on hearing function or other extra-pancreatic features.

Five of the 15 participants for whom follow-up data on thiamine treatment was collected are currently aged 13 years or older. All of them have achieved normal puberty and are still transfusion-independent. These five patients are insulin-dependent and three had no improvement in their initial poor diabetes response to thiamine.

There were no reported side effects of thiamine therapy. One individual (9.1) had a stroke at the age of 2 years while on combined insulin and thiamine therapy. Individual 10.1 died at the age of 7 years. He had diabetes diagnosed at the age of 2 years and was initially treated with insulin. Subsequently thiamine was added and his glycaemic control improved during the following 4 years. However, he died of DKA following a viral illness during a family holiday to a remote area. Individual 27.1 developed hyperglycaemia during intercurrent illnesses while she was off thiamine therapy.

## Discussion

Early diagnosis of TRMA syndrome is important to allow targeted treatment, as more than half of the individuals with follow-up data benefited from early treatment with thiamine, with some individuals becoming insulin-independent. At high concentrations, thiamine can cross the cell membrane through passive diffusion and thus individuals with TRMA syndrome, who lack functional THTR1, can respond to supraphysiological thiamine doses. However, before our study, data on the impact of thiamine therapy, particularly on diabetes, was limited. We describe the largest series of individuals with TRMA syndrome collected to date, with long-term follow-up data for 15 patients on thiamine therapy.

Thiamine therapy improved diabetes control in more than 70% of participants (11/15) with four individuals managing to stop insulin treatment. Our data suggest that the best predictive factors for good glycaemic response to thiamine therapy were early diabetes diagnosis, early referral for genetic testing and early initiation of thiamine treatment, with 10 of the 11 participants showing improvement in glycaemic control having started thiamine before the age of 3 years. These results highlight the importance of genetic testing to deliver targeted therapy in this subtype of diabetes, as previously suggested for other neonatal diabetes subtypes [24]. The exact dose of thiamine required to achieve clinical benefit is unknown; however, in this cohort, there seemed to be no additional benefit for thiamine doses >150 mg/day. Because HbA_1c_ is largely dependent on red cell survival/turnover, which is affected by thiamine deficiency, it is possible that the thiamine replacement can lead to a reduction in HbA_1c_ as a result of rapid red cell turnover. However, in this cohort, Hb normalised in all individuals treated with thiamine but only 11/15 had a reduction in HbA_1c_ and a lower insulin requirement and four of them were able to stop insulin. This suggests that the reduction in HbA_1c_ is likely to be a true reflection of improved glycaemic control following thiamine therapy. Sadly, one individual in the responders group died of DKA at 7 years old during a holiday. Although the cause of death was attributed to improper management in a remote setting, it is important to alert parents that, as with other children with diabetes, individuals with TRMA syndrome can develop DKA and to educate them on sick days management and early recognition of DKA symptoms.

Similar to previous reports [15, 17], anaemia and thrombocytopenia resolved in all participants treated with thiamine, but deafness persisted. The variable impact of thiamine therapy on anaemia, diabetes and deafness may reflect a variation in the sensitivity to thiamine deficiency between different tissues. Previous reports [15, 27] have indicated that thiamine responsiveness decreases after puberty and that adults with TRMA syndrome may become transfusion-dependent and require insulin treatment despite previously adequate thiamine supplementation. We did not observe this in participants of pubertal and post-pubertal age in our cohort and it was not reported in two Italian individuals with TRMA syndrome followed for 20 years [17]. However, one of the individuals in our cohort who became insulin-independent (20.1) has recently started to require insulin again at the age of 11 years. Further follow-up investigations will be needed to assess changes in thiamine sensitivity at different ages.

All the participants in our cohort were diagnosed with diabetes, with approximately 50% of them developing diabetes in the first year of life. The high incidence of very early onset diabetes in our cohort could be partly due to referral bias, since 31/32 participants were tested by the Exeter genetics laboratory as part of a research study on monogenic diabetes. Diabetes is, however, a cardinal feature of TRMA syndrome and it can precede the appearance of other features, as was observed in three individuals in our cohort. Using a targeted next generation sequencing assay we were able to provide a genetic diagnosis of TRMA syndrome in two children with isolated early-onset diabetes. In one of them (19.1) the genetic diagnosis led to starting thiamine treatment when she had isolated neonatal diabetes and the individual is currently off insulin, yet to develop anaemia and has started using hearing aids following a hearing test. This case highlights the benefits of a pharmacogenetic approach in management of individuals with this subtype of diabetes.

A clinical diagnosis of TRMA syndrome is usually based on the presence of diabetes, deafness and anaemia. However, waiting for all three features to develop may lead to a delay in obtaining the diagnosis and starting the appropriate treatment. Our data show that only three individuals presented with the full triad of symptoms, whilst all the others presented with one or two of the cardinal features. We suggest that a diagnosis of TRMA should be considered in children with isolated diabetes presenting in the first year of life and that a normal Hb and/or MCV should not preclude genetic testing for mutations in *SLC19A2*. Interestingly, unlike type 1 diabetes, some participants in our cohort were incidentally found to have asymptomatic hyperglycaemia, indicating that some of these individuals may have a long period before diabetes symptoms appear. Therefore, it would be useful to do a routine blood glucose or HbA_1c_ measurement in individuals with the combination of anaemia and deafness.

Although this observational study has been conducted on the largest cohort of individuals with TRMA syndrome collected to date, our cohort is relatively small and follow-up data was available for only half of the participants. This is an important limitation of our study and further investigations in larger cohorts will be necessary to confirm our findings. Furthermore this also means that we could only follow up participants who were still alive and this could be introducing a bias in our analysis towards the thiamine-responsive group. Again, further studies in larger cohorts will be needed to overcome this limitation. Since this is an international cohort of individuals diagnosed with TRMA syndrome over a period of >10 years, the response to thiamine was collected retrospectively and participants were managed in different centres using different protocols. A prospective study using similar doses, with measurement of C-peptide and other markers of beta cell reserve would probably further our understanding of the pathophysiology of this subtype of diabetes and the role of thiamine in the management of this condition in individuals with TRMA syndrome.

In summary, *SLC19A2* mutations are a rare cause of diabetes which can present without anaemia and deafness. A genetic diagnosis of TRMA syndrome is fundamentally important to guide clinical management, since exogenous thiamine therapy has been shown to reverse some of the clinical features of the disease.

## Electronic supplementary material

ESM (PDF 310 kb)

## Data Availability

*SLC19A2* mutation details have been deposited in the Decipher database (https://decipher.sanger.ac.uk/).

## Abbreviations

DKA: Diabetic ketoacidosis
Hb: Haemoglobin
IQR: Interquartile range
MCV: Mean corpuscular volume
THTR1/THTR2: Thiamine Transporter 1/2
TRMA: Thiamine-responsive megaloblastic anaemia
