# Supplementary material for: Pharmacogenomics in diabetes: outcomes of thiamine therapy in TRMA syndrome
Source: Diabetologia. 2018 Feb 15;61(5):1027–36. doi: 10.1007/s00125-018-4554-x (PMC6449001; doi:10.1007/s00125-018-4554-x)
Supplement: Supplementary file 1 — (PDF 310 kb) [file 125_2018_4554_MOESM1_ESM.pdf]

ELECTRONIC SUPPLEMENTARY MATERIAL

International Neonatal diabetes consortium:

V Iotova (University Hospital 'St. Marina', Varna, Bulgaria), J Raza (National Institute of Child Health, Karachi, Pakistan), J Grulich-Henn (University Children´s Hospital, University of Heidelberg, Heidelberg, Germany), K Matyka (Division of Metabolic and Vascular Health, Warwick, UK), IC Verma (Institute of Medical Genetics and Genomics, Sir Ganga Ram Hospital, New Delhi , India), I Perthus (Service de Génétique Médicale, CHU de Clermont-Ferrand, Clermont-Ferrand, France), R Casey (Alberta Health Services, Edmonton, Canada), P Taberner (Section of Nutrition and Diabetes, Children's General Hospital "Dr. Pedro de Elizalde", Buenos Aires, Argentina), R Bundak (Istanbul Faculty of Medicine, Department of Pediatrics, Pediatric Endocrinology Unit, Istanbul University, Istanbul, Turkey), A Malik (Frimley Children's Centre, Frimley, Surrey, UK), F Adriana (Federico II University Hospital, Napoli, Italy), S Soto (Department of Pediatrics, Roosevelt Hospital, 5ta Avenida Calzada Roosevelt, Zona 11, Guatemala City, Guatemala). M Hassan (Department of Pediatrics, Cairo university, Cairo, Egypt), R El-Kaffas (clinical and chemical pathology department, Cairo University, Egypt), A Abdelkader (Department of Pediatrics, Cairo University, Cairo, Egypt).

Supplement table 1: demography and detailed genotype of the cohort

Patient	Country	Consanguinity	Gender	*SLC19A2* mutation (Novel mutations in bold)			
				Exon	Mutation type	Mutation name	Nucleotide change
1.1	Pakistan	Yes	Male	**1**	**Missense**	**p.L64P/p.L64P**	**c.191T>C/c.191T>C**
1.2	Pakistan	Yes	Female	**1**	**Missense**	**p.L64P/p.L64P**	**c.191T>C/c.191T>C**
1.3	Pakistan	Yes	Male	**1**	**Missense**	**p.L64P/p.L64P**	**c.191T>C/c.191T>C**
2.1	Pakistan	Yes	Female	**1**	**Nonsense**	**p.W30X/p.W30X**	**c.89G>A/c.89G>A**
3.1 [16]	England	Yes	Female	1	Nonsense	p.E66X/p.E66X	c.196G>T/c.196G>T
4.1	India	No	Female	1	Nonsense	p.E66X/p.E66X	c.196G>T/c.196G>T
5.1 [16]	Germany / Kurdi Iraq	Yes	Male	2	Nonsense	p.Y79X/p.Y79X	c.237C>A/c.237C>A
6.1 [20]	Italy	No	Female	2/6	Nonsense	p.Y81X/p.L457X	c.242dup/c.1370del
6.2 [20]	Italy	No	Female	2/6	Nonsense	p.Y81X/p.L457X	c.242dup/c.1370del
7.1	Argentina	No	Female	**2**	**Missense**	**p.A91P/p.A91P**	**c.271G>C/c.271G>C**
8.1	India	Yes	Female	2	Missense	p.G105E/p.G105E	c.314G>A/c.314G>A
9.1 [16]	Sudan	Yes	Male	2	Frameshift	p.I109fs/p.I109fs	c.327_334del/c.327_334del
10.1	Sudan	Yes	Female	2	Frameshift	p.I109fs/p.I109fs	c.327_334del/c.327_334del
10.2	Sudan	Yes	Female	2	Frameshift	p.I109fs/p.I109fs	c.327_334del/c.327_334del
11.1 {Mikstiene, 2015 #293	Saudi Arabia	Yes	Female	2	Missense	p.S143F/p.S143F	c.428C>T/c.428C>T
12.1	France	No	Female	2	Missense	p.S143F/p.S143F	c.428C>T/c.428C>T
13.1	Canada	No	Male	2	Frameshift	p.G152X/p.G152X	c.454_458delGGCATinsTA/c.454_458delGGCATinsTA
14.1	Honduras	No	Male	2	Missense	p.G172D/p/G172D	c.515G>A/c.515G>A
15.1	Turkey	Yes	Male	2	Frameshift	p.S214fs/p.S214fs	c.641del/c.641del
16.1 [26]	England/iran	Yes	Female	2	Nonsense	p.Q233X/p.Q233X	c.697C>T/c.697C>T
17.1 [26]	England/ Iran	No	Male	2	Nonsense	p.Q233X/p.Q233X	c.697C>T/c.697C>T
17.2 [26]	England/ Iran	No	Female	2	Nonsense	p.Q233X/p.Q233X	c.697C>T/c.697C>T

18.1 [26]	England/ Iran	Yes	Male	2	Nonsense	p.Q233X/p.Q233X	c.697C>T/c.697C>T
19.1	Honduras	No	Female	2	Nonsense	p.E254X/p.E254X	c.759dupT/c.759dupT
20.1	KSA	Yes	Female	**3**	**Nonsense**	**p.W302X/p.W302X**	**c.905G>A/c.905G>A**
21.1	England	No	Male	3	Missense	p.W320G/p.W320G	c.958T>G/c.958T>G
22.1	Bulgaria	No	Female	**3**	**Frameshift**	**p.N333fs/p.N333fs**	**c.993_996dup/c.993_996dup**
23.1	Czech Republic	No	Female	**1/3**	**Splicing/Missense**	**p.?/p.G334D**	**c.204+2T>G/c.1001G>A**
24.1 [16]	Czech Republic	No	Female	3/4	Missense/ Frameshift	p.G334D/p.V383fs	c.1001G>A; c.1148_1149del
25.1 [22]	India	Not confirmed	Female	3	In-frame deletion	p.G335del/p.G335del	c.1002_1004del/c.1002_1004del
26.1	Guatemala	Not confirmed	Female	**4**	**Frameshift**	**p.M401fs/p.M401fs**	**c.1201_1202del/c.1201_1202del**
27.1	Egypt	Yes	Male	4	**Nonsense**	**p.W387x/ p.W387x**	**c.1160G>A/c.1160G>A.**
